# OCRFinder: a noise-tolerance machine learning method for accurately estimating open chromatin regions

**DOI:** 10.3389/fgene.2023.1184744

**Published:** 2023-06-01

**Authors:** Jiayi Ren, Yuqian Liu, Xiaoyan Zhu, Xuwen Wang, Yifei Li, Yuxin Liu, Wenqing Hu, Xuanping Zhang, Jiayin Wang

**Affiliations:** ^1^ School of Computer Science and Technology, Xi’an Jiaotong University, Xi’an, China; ^2^ Shaanxi Engineering Research Center of Medical and Health Big Data, Xi’an Jiaotong University, Xi’an, China

**Keywords:** cell-free DNA - cfDNA, open chromatin region, noisy label learning, chromatin accessibility, sequencing data analyses

## Abstract

Open chromatin regions are the genomic regions associated with basic cellular physiological activities, while chromatin accessibility is reported to affect gene expressions and functions. A basic computational problem is to efficiently estimate open chromatin regions, which could facilitate both genomic and epigenetic studies. Currently, ATAC-seq and cfDNA-seq (plasma cell-free DNA sequencing) are two popular strategies to detect OCRs. As cfDNA-seq can obtain more biomarkers in one round of sequencing, it is considered more effective and convenient. However, in processing cfDNA-seq data, due to the dynamically variable chromatin accessibility, it is quite difficult to obtain the training data with pure OCRs or non-OCRs, and leads to a noise problem for either feature-based approaches or learning-based approaches. In this paper, we propose a learning-based OCR estimation approach with a noise-tolerance design. The proposed approach, named OCRFinder, incorporates the ideas of ensemble learning framework and semi-supervised strategy to avoid potential overfitting of noisy labels, which are the false positives on OCRs and non-OCRs. Compared to different noise control strategies and state-of-the-art approaches, OCRFinder achieved higher accuracies and sensitivities in the experiments. In addition, OCRFinder also has an excellent performance in ATAC-seq or DNase-seq comparison experiments.

## 1 Introduction

Open chromatin regions (OCRs) are the particular regions associated with cellular physiological activities. They exposed when highly folded chromatin structures are replicated and transcribed, which can bind to intranuclear macromolecules regulated by DNA regulatory elements (Flavahan et al., 2017; Klemm et al., 2019; Minnoye et al., 2021). Chromatin accessibility in cancer provides a link between gene expression and somatic mutations, DNA methylation, and distant regulatory elements (Corces et al., 2018; Wang et al., 2019; Shin et al., 2021). For example, in gastric cancer, by estimating corresponding OCRs, researchers can significantly distinguish anti-PD-1 therapy responders from non-responders (Shin et al., 2021). Moreover, since OCRs exhibit different patterns in different cancer species, the estimation of OCRs can also be used to predict tumor markers and analyze the epigenetic mechanisms of cancer (Jiang et al., 2018; Sun et al., 2019; Ulz et al., 2019; Wang et al., 2019).

**FIGURE 1:**
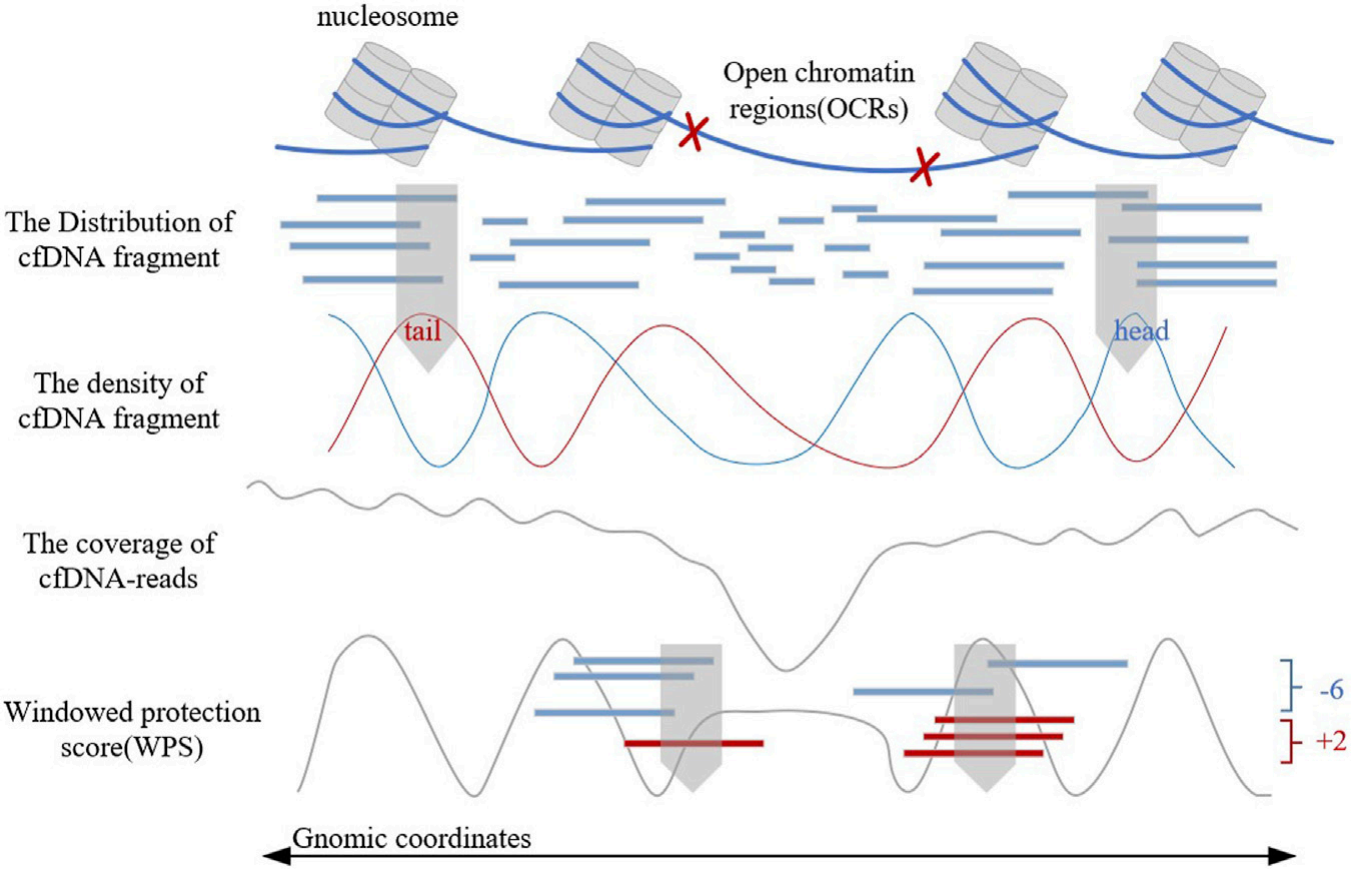
The distribution and characteristics of cfDNA fragments.

Traditional OCR estimating method relies on tissue sampling, which is a complex experimental process. In recent years, the discovery of the relationship between cell-free DNA (cfDNA) fragment characteristics and gene expression levels (Snyder et al., 2016; Ulz et al., 2016) has provided a more convenient, comprehensive, and safer method for OCR estimation (Sun et al., 2019; Wang et al., 2021). These methods analyze the biological background of OCRs, observe the distribution of cfDNA fragments, construct corresponding features artificially, and combine with machine learning classifiers to finally achieve the estimation of OCRs. Figure 1 shows the distribution of cfDNA fragments and related cfDNA-features in OCRs. These artificially constructed features could reflect the distribution of cfDNA fragments to a certain extent. But when the cfDNA-seq data has low sequencing depth or gene mutations exist, these features will be disturbed and have limitations. In addition, these manually constructed features are subjective and can hardly give a good picture of cfDNA distribution. Therefore, it is not enough for artificially constructed features, and a more objective feature construction way is needed. However, there is no current method for OCR estimation with automatic feature extraction, such as LDA (Campbell et al., 2015) and neural networks.

Moreover, the labels used by these methods are based on statistical rules, such as considering regions with active gene expression as OCRs and regions with silent gene expression as closed chromatin regions (CCRs). Thus there is a potential problem: labels used for OCR estimation are in error. Due to the individual specificity of biological samples, the chromatin accessibility in the same region can vary from individual to individual and from time to time (Wang et al., 2019). Even if the experimental means of ATAC-seq (Buenrostro et al., 2015) and DNase-seq (Natarajan et al., 2012) are used to validate labels, the dynamic change problem of chromatin accessibility cannot be solved perfectly, not to mention its economic, time, and sampling burden. Thus, how to address the inevitable noisy labels in open chromatin regions becomes the key to the OCR estimation problem.

Therefore, in this paper, we propose a novel OCR estimation algorithm called OCRFinder. It is centered on avoiding or mitigating the interference of noisy labels. It combines the characteristics of cfDNA-seq data and the relationship between OCR and gene expression levels to perform noisy label learning in OCRs by ensemble ideas and semi-supervised strategies.

## 2 Background

### 2.1 The estimation of OCRs

The traditional OCR estimation methods (ChIP-Seq (Lorzadeh et al., 2016), ATAC-seq, DNase-seq, and Mnase-seq (Mieczkowski et al., 2016)) surgically obtain tissue cells and culture them *in vitro*. Then these methods use specific enzymes to cleave DNA sequences and obtain exposed OCRs that can bind to regulatory factors. Although reliable, these methods are invasive biopsies, which are complex, harmful, and difficult to apply to clinical applications.

Plasma cfDNA is derived from apoptotic cells and can be obtained by safe and convenient liquid biopsy (Snyder et al., 2016). With the discovery of cfDNA fragment characteristics in OCRs, chromatin accessibility studies through cfDNA-seq data have gradually become popular.

In 2016, Snyder first found that the distribution of cfDNA fragments could reflect the distribution of nucleosomes and proposed the windowed protection score (WPS) algorithm to present it digitally (Snyder et al., 2016). The WPS waveform can reflect the position of nucleosomes and provide support for OCR estimation. In the same year, Ulz proposed EP (Expression Prediction) algorithm (Ulz et al., 2016), which focused on the low sequencing coverage in OCRs to estimate the gene expression levels. OCRs are associated with gene expression, but the EP algorithm cannot directly identify them. In 2019, Lo (Sun et al., 2019) proposed that the differentially phased fragment end signals could reflect the characteristics of cfDNA fragment distribution. Based on the WPS waveform and sequencing coverage, Wang proposed the OCRDetector algorithm in 2021 (Wang et al., 2021). It is the first OCR estimation algorithm in whole genome regions through cfDNA-reads, but it could hardly solve the limitations of artificially constructed features.

In this paper, deep neural networks will be used for feature extraction as well as classification. As long as an effective encoding method is provided, deep learning models can automatically extract the characteristic fragmentation pattern of cfDNA molecules at OCRs without manual intervention. Therefore, using deep learning for OCR estimation is a feasible approach.

### 2.2 The method of noisy label learning

Most existing noisy label learning methods can be classified into two main categories. The first type is to design noise-resistant loss functions by exploiting the characteristics of noisy data to correct the gradients of noisy samples or to balance their negative effects (Arazo et al., 2019; Han et al., 2019; Liu et al., 2020; Ma et al., 2020). However, these methods tend to depend on the *a priori* information about the data and have application limitations (Wang et al., 2022). Another category is to design a sample selection strategy to separate clean samples from noisy samples (Reed et al., 2014). A common approach is to consider samples with fewer losses as clean samples (Shen and Sanghavi, 2019). Jiang (Jiang et al., 2304) used a data-driven curriculum learning strategy to solve the noisy-label-overfitting problem. Ren (Ren et al., 2018) assigns different weights to the samples by the meta-learning approach. Chen (Chen et al., 1062) proposes a method to clean the data using a cross-validation method. Han (Han et al., 2018) used the idea of co-training to train two networks to improve the noisy label overfitting problem. Yu (Song et al., 2019) updated it by using disagreement of the co-trained networks to maintain the robustness of the model to the noise labels. But these methods rely on the data prior information and cleaning guidelines. For OCR estimation in the biomedical field, such *a priori* information is difficult to obtain, so this paper proposes a division method without *a priori* information and integrates integration and semi-supervised ideas to improve the robustness of the model.

## 3 Materials and methods

**FIGURE 2:**
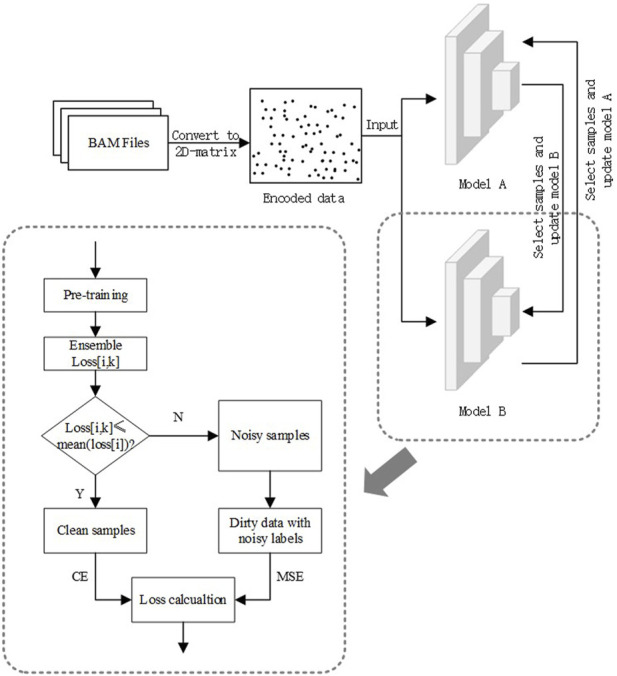
Algorithm flow chart.

To solve the problem of estimating OCRs with noisy labels, we propose a three-stage binary classification algorithm that introduces the ideas of ensemble learning as well as semi-supervised learning into it. The three stages are data pre-processing, model pre-training, and model semi-supervised training. The last step consists of three parts: sample selection, calculation of ensemble loss, and recirculation of dirty samples. The data pre-processing converts cfDNA-seq data to two-dimensional images. The second stage gives the model an initial discriminatory capability and requires the design of a loss function with noise-resistant characteristics to avoid early overfitting of noisy data. In the last stage, we design a reliable and adjustable division criterion and use semi-supervised ideas to balance data utilization and training noise rate. The general flow chart of the algorithm is shown in Figure 2.

### 3.1 Data pre-processing

First, the cfDNA-seq data in fastq format is processed by BWA and Samtools to obtain the cfDNA-reads data in bam format. Then, OCRFinder encodes cfDNA-reads data into two-dimensional matrixes *T*, rows representing genomic coordinates and columns representing cfDNA-reads lengths. For example, *T*_*ij*_ denotes the number of cfDNA-reads with genomic coordinate *i* and length *j*. Besides, We only consider the cfDNA-reads with lengths between 50 bp-250 bp.

In addition, since sequencing coverage, WPS score, and the density of the head and tail of cfDNA fragments can help reflect the gene expression, we encode these four artificial features in the same way, to obtain two-dimensional matrixes as another input to OCRFinder.

### 3.2 Model pre-training

**FIGURE 3:**
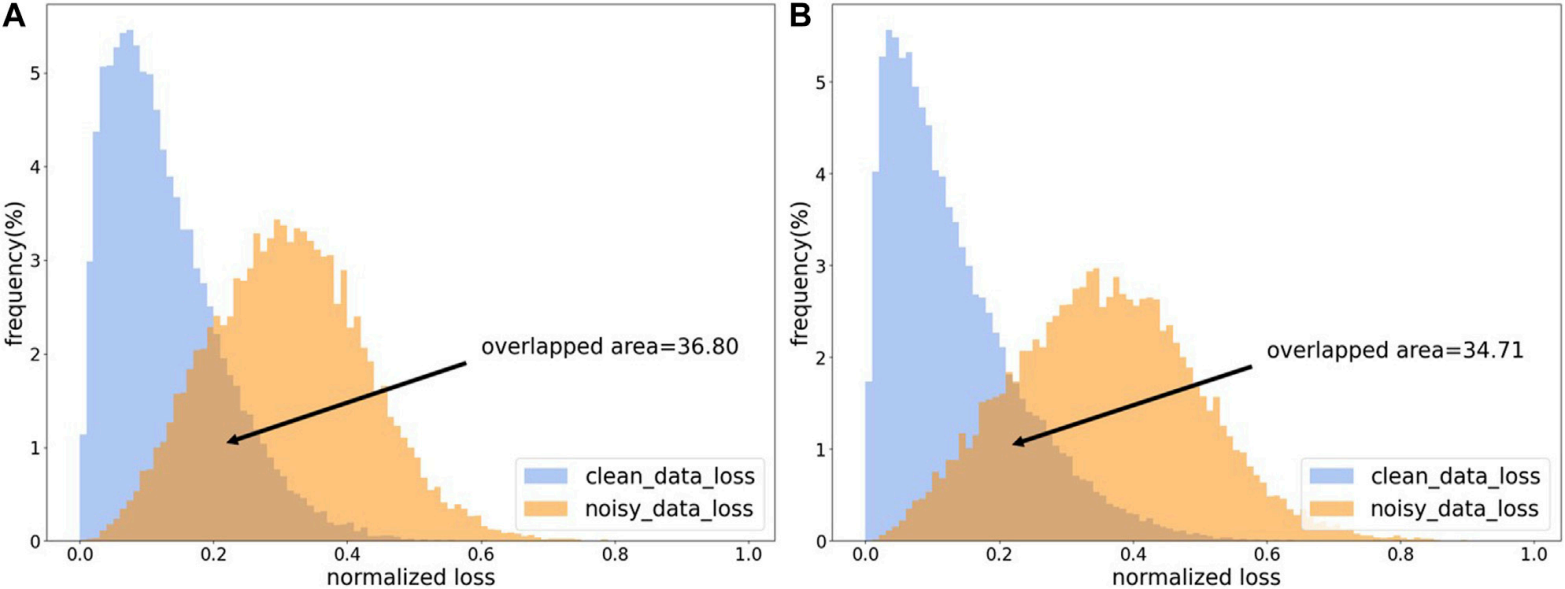
The loss distribution map. **(A)** The loss distribution obtained from CE loss function. **(B)** The loss distribution obtained by CE loss function with L1 regulation.

Most of the sample selection methods perform data cleaning based on the losses of samples. Because the model will first fit clean samples during iterative training, the losses of clean samples will be smaller than that of noisy samples (Han et al., 2018). As shown in Figure 3, the losses of clean samples and the losses of noisy samples show two different distributions. In order to make the model have an initial discriminative ability in the training process, an additional pre-training session is needed, which is also to make the model better adapted to the learning rate and other settings. At the same time, the pre-training phase needs to be constrained to avoid overfitting noisy labels during the pre-training period.

The cross-entropy (CE) loss is the most common classification loss function. Still, it is prone to the overfitting problem of noisy data, interfering with subsequent data cleaning. It has been found that symmetric loss functions can tolerate noisy labels, and the mean absolute error (MAE) loss is one of them. But MAE loss trains slowly and is difficult to converge (Ma et al., 2020). As shown in Supplementary Figure S1, as the number of iterations increases, there will be a significant drop in the test accuracy of CE loss after reaching the peak.

In contrast, the test accuracy of MAE loss will slowly increase, and in the training set, CE loss is much easier to fit. So, the combination of the binary cross-entropy (BCE) function and mean absolute error function in this paper is used as the pre-trained loss function; that is, the BCE loss is bounded by L1 regularization (Equation 1). Improving the loss function not only ensures the stability of the model, but also increases the comprehensibility of the machine learning model (Wang et al., 2023).

In addition, because the number of different OCRs varies greatly, we also studied the relationship between the size of the training set and the performance of CE loss and MAE loss. And the experimental results show that as the training set increases, CE loss will be subject to noise labeling and lead to performance degradation, while MAE loss is difficult to train when the training set is small (Supplementary Figure S2). Therefore, according to the size of the training set, we designed a weighting factor to regulate the percentage of CE loss and MAE loss (Equation 2).

$$\begin{array}{l} Loss=CE\left(x,y\right)+\psi MAE\left(x,y\right)\\ =-y\log p\left(y|x,\theta \right)+\left(1-y\right)\log(1-p\left(y|x,\theta \right)+\psi \|p\left(y|x,\theta \right)-y\|, \end{array}$$ (1)

$$\psi \left(n\right)={\left(\frac{n}{t}\right)}^{\gamma },$$ (2)

where $x\in R^{m\times n}$ is the two-dimensional input, $y\in \left\{0,1\right\}$ is the corresponding label, θ is the trained network parameter, n is the size of the training set, t is the fixed size taken as 1,000, and γ is the adjustment factor taken as 0.5 in this paper.

As shown in Figure 3, our proposed regularized CE loss will effectively alleviate the loss-overlap areas between noisy samples and clean samples. The L1 regularized CE loss can somewhat reduce the overfitting of noisy labels.

In addition, our OCR dataset only contains unknown and noisy labels. To illustrate the noisy-label influence on CE loss, MAE loss, and sample loss distributions, here we used the CIFAR10 dataset with exact labels and artificially assigned noisy labels to this dataset.

### 3.3 Model semi-supervised training

#### 3.3.1 Sample selection

With an initial discriminative power, the model will be used for data cleaning. It has been shown that the network tends to remember clean samples more than noisy ones in the initial stage of model training. As the number of iterations increases, the network gradually fits the noisy samples. Eventually, the network will remember all the samples ideally, showing that the losses of clean samples will be smaller than those of noisy samples (Huang et al., 2019). Therefore, most sample selection methods select data based on the magnitude of sample loss. However, in many sample selection methods, the thresholds for data cleaning will rely on prior information, which is not present in the OCR estimation problem. Therefore, OCRFinder will use the average of the sample losses as a dynamically adjustable division threshold (Equation 4). Samples with Losses smaller than the threshold are considered clean, and *vice versa* are noisy. The losses are calculated based on the BCE function. For the *k*th sample, its loss is:

$$loss_{k}=-\left(y_{k}\cdot \log\left(p\left(y_{k}\right)\right)+\left(1-y_{k}\right)\cdot \log\left(1-p\left(y_{k}\right)\right)\right),$$ (3)

Where ${loss}_{k}$ is the loss value of the *k*th sample, *k* is the sample number, $y_{k}$ is the sample label, and $p\left(∙\right)$ is the model prediction.

Unlike existing methods, we draw on confident learning (Northcutt et al., 2021) and take the mean loss of samples as the threshold to adjust the threshold adaptively during the iterative process. The threshold for the *k*th sample is:

$$\tau_{y_{k}}=\frac{1}{K}\sum_{k=1}^{K}loss_{k}^{y_{k}},$$ (4)

where $y_{k}$ is the sample label, $\tau_{y_{k}}$ is the division threshold of the samples with category $y_{k}$ , and K is the number of samples with labeled label $y_{k}$ .

Unlike existing methods, we draw on the idea of confidence learning and use the mean loss of the samples as the threshold in order to adjust the threshold adaptively during the iterative process.

#### 3.3.2 Calculation of loss function

It has been found that while training a dataset with noisy labels, the model will forget the noisy samples more quickly than the clean samples (Toneva et al., 2018). In other words, the noisy samples’ losses will be unstable in the iteration process. Thus, the loss for each iteration of each sample during training can be recorded, and the noise sample can be judged based on the number of individual forgotten events and the variance of the individual sample loss. However, this approach will lead to high spatial complexity and challenge the experimenter’s equipment conditions. Therefore, we adopt an exponential moving average strategy for loss calculation and integrate the historical losses with the current losses to obtain more reliable cleaned samples. Their loss values were calculated as follows:

$$Loss_{i,k}=\alpha Loss_{i-1,k}+\left(1-\alpha \right)Loss_{i,k},$$ (5)

Where *i* represents the number of iterations, *k* represents the sample number, ${Loss}_{i,k}$ represents the loss of the *kth* sample in the *ith* iteration, α is the moving average coefficient, which can be adjusted for different occasions, and in this paper, α is 0.3 in all experiments. In this way, the loss for the current iteration depends on its current forward propagation calculation and the losses obtained in the previous iteration, thus utilizing the historical information of the sample loss.

#### 3.3.3 Recirculation of dirty samples

To further mitigate the noise label overfitting problem, we adopt the idea of co-training: two identical models are built, and in each iteration, each cleaned data is selected to guide each other in the back-propagation of the model. For example, in the *k*th iteration, model *A* obtains a clean dataset *D*_*A*_ in the forward propagation computation, and then model *B* uses *D*_*A*_ to backpropagate and update itself; conversely, the same is true for the update of model *A*. In this way, the overfitting problem caused by a single model using the same parameters for both prediction and update can be indirectly avoided.

To make full use of the training set, we apply the semi-supervised idea. It uses the cleaned dataset for back-propagation and assigns pseudo-labels (Equation 7) to samples from the noisy dataset for semi-supervised training. Moreover, we also designed a sharpen function to sharpen pseudo-labels (Equation 6). The sharpening operation can enhance the effect of samples with consistent model prediction and weaken the impact of samples with disagreement model prediction.

$$Sharpen\left(x\right)=\frac{e^{\frac{x}{\tau }}}{e^{\frac{x}{\tau }}+e^{\frac{1-x}{\tau }}},$$ (6)

Where *τ* is the sharpening factor, the smaller is *τ*, the more obvious sharpening effect is (Supplementary Figure S3). In this paper, The value of *τ* is 0.3.

Here, we adopt the same strategy for the sample confidence as for the integration loss, and the confidence for the *k*th sample in the *i*th iteration is:

$$label_{k}={\mathrm{argmax}}_{c}\left(Sharpen\left(p\left(y_{k,A}\right)\right)+Sharpen\left(p\left(y_{k,B}\right)\right)\right)/2,$$ (7)

$$p_{i}\left(y_{k,A}\right)=\alpha \cdot p_{i}\left(y_{k,A}\right)+\left(1-\alpha \right)\cdot p_{i-1}\left(y_{k,A}\right),$$ (8)

$$p_{i}\left(y_{k,B}\right)=\alpha \cdot p_{i}\left(y_{k,B}\right)+\left(1-\alpha \right)\cdot p_{i-1}\left(y_{k,B}\right),$$ (9)

Where ${label}_{k}$ represents the pseudo label for the *k*th sample, $y_{k,A}$ is the prediction obtained by model A, $y_{k,B}$ is the prediction obtained by model *B*, α is the same as in Equation 5.

For this part of the loss calculation, we focus more on the consistency of the sample and use the mean square error loss function (Equation 10). For clean samples, we use the common BCE loss function (Equation 11).

$$Loss_{d}=\frac{1}{M}\cdot \sum_{m=1}^{M}{\left\|p\left(y_{m}\right)-label_{m}\right\|}^{2},$$ (10)

$$Loss_{c}=-\frac{1}{N}\cdot \sum_{n=1}^{N}y_{n}\cdot \log\left(p\left(y_{n}\right)\right)+\left(1-y_{n}\right)\cdot \log\left(1-p\left(y_{n}\right)\right),$$ (11)

Where ${label}_{m}$ represents the pseudo label for the *m*th sample in noisy samples*, M* is the number of noisy samples, and *N* is the number of clean samples.

Therefore, the final loss function is:

$$Loss=Loss_{c}+Loss_{d}.$$ (12)

**FIGURE 4:**
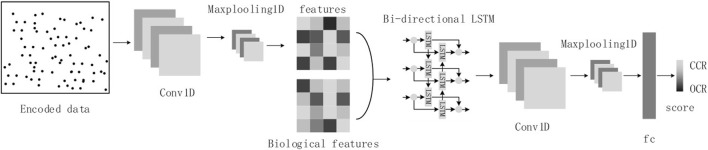
The model structure diagram.

Considering the sequence nature of cfDNA-reads and the image nature of cfDNA fragment distribution in OCRs, we will refer to the structure of the DanQ model (Quang and Xie, 2016), where we transform the cfDNA-seq data to images as input. The model consists of a one-dimensional convolutional layer, a max-pooling layer, a bidirectional LSTM layer, a one-dimensional convolutional layer, a max-pooling layer, and a fully connected layer (Figure 4).

Moreover, since our focus is not on feature extraction but on how to design a noisy label learning algorithm based on deep learning for OCR estimation, this paper does not care about the selection of deep learning models.

## 4 Results

We use the same approach as OCRDetector for evaluating our algorithm’s performance based on the known gene expression levels or the chromatin accessibility levels. For example, as many OCRs as possible for housekeeping genes should be detected due to their high expression, while for some low-expression genes, fewer OCRs should be detected. We use the index of sensitivity for evaluation (Equation 13). *TP* (True Positive) represents the number of samples with OCRs labeled as positive, and *FN* (False Negative) represents the number of samples with closed chromatin regions labeled as positive. Besides, we also use AUC and AURP metrics to evaluate the model’s performance. AUC is the area under the ROC curve, and AUPR is the area under the PR curve. Both of them indicate the classification ability of the model.

$$Sensitivity=\frac{TP}{TP+FN}.$$ (13)

The dataset used in this paper is provided by (Snyder et al., 2016) and stored in the National Center for Biotechnology Information (NCBI) database with the accession number SRR2130051, which is from a healthy population with an average sequencing depth of 20x. Although the number of individual samples for cfDNA-seq of the dataset is small, it is still sufficient for training deep learning models. Because each sample contains tens of chromosomes, the dataset used in this paper is substantial, and the number of available training samples is in the thousands.

Since chromatin accessibility is related to gene expression levels, when a gene region is in the state of transcription or replication, its nucleosome will be shed, and this chromatin region will be in the open state. Therefore, for the training set, we select the highly expressed housekeeping genes as the positive samples and non-genetic regions as the negative samples because gene expressions are absent in non-genetic regions. It should be noted that since chromatin accessibility is dynamically changing, positive samples will inevitably contain negative samples labeled as positive, and negative samples will also have noisy labels.

Our training samples are the 2-kbp regions around the gene transcription start site and any 2-kbp regions in the non-genetic regions. Chromosomes 2-7 are used for training, and chromosome 1 is used for testing. We keep the number of positive and negative samples the same. The amount of the training set is 2,144.

For test set, we also selected highly expressed housekeeping genes as positive samples and non-genetic regions without gene expression as negative samples. In addition, to exclude gene-specific chance, we also combined the results of ATAC-seq and DNase-seq experiments and calculated the overlap percentage of OCRs obtained by OCRFinder and ATAC-seq or DNase-seq experiments in hematopoietic lineage cells. This overlap percentage represents the sensitivity of OCRFinder on the OCRs from ATAC-seq or DNase-seq experiments. We conducted comparison experiments on three positive test sets: HK_TSS, Hematopoietic_Lineage_ATAC_OCRs, Hematopoietic_lineage_DNase_OCRs, and one negative test set Non_Genetic_Regions. Although there is still some noise in the test set, metrics such as sensitivity, AUC, and AUPR can also be used to evaluate the model’s performance as long as the results of the model on the test set are reasonable.

We used ConvLSTM network and Adam optimizer. The learning rate was 1e-4, the batch size was 128, the training epoch was 150, and the warm-up training epoch was 10. This paper’s results are the average of five random experiments, each with a different random seed.

### 4.1 Analysis of sensitivity and false positives compared with OCRDetector

**FIGURE 5:**
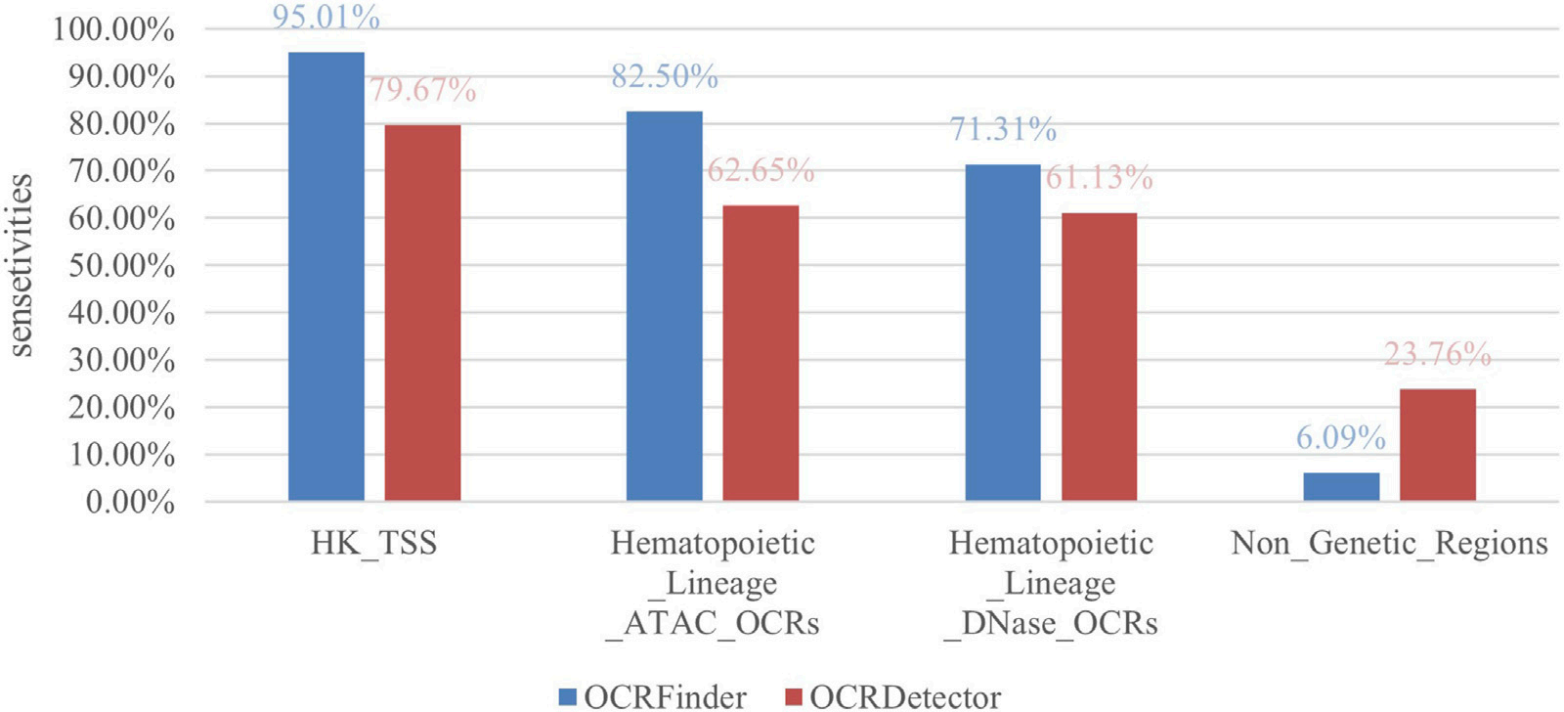
Sensitivity comparison between OCRFinder and OCRDector using different test sets.

We compared our method with OCRDetector, and as shown in Figure 5, our method had higher sensitivity in regions with housekeeping genes and hematopoietic lineage cells. And our method detected a much lower percentage of OCRs in non-genetic regions than OCRDetector, indicating that our method is able to suppress false positives while detecting more true positives.

**FIGURE 6:**
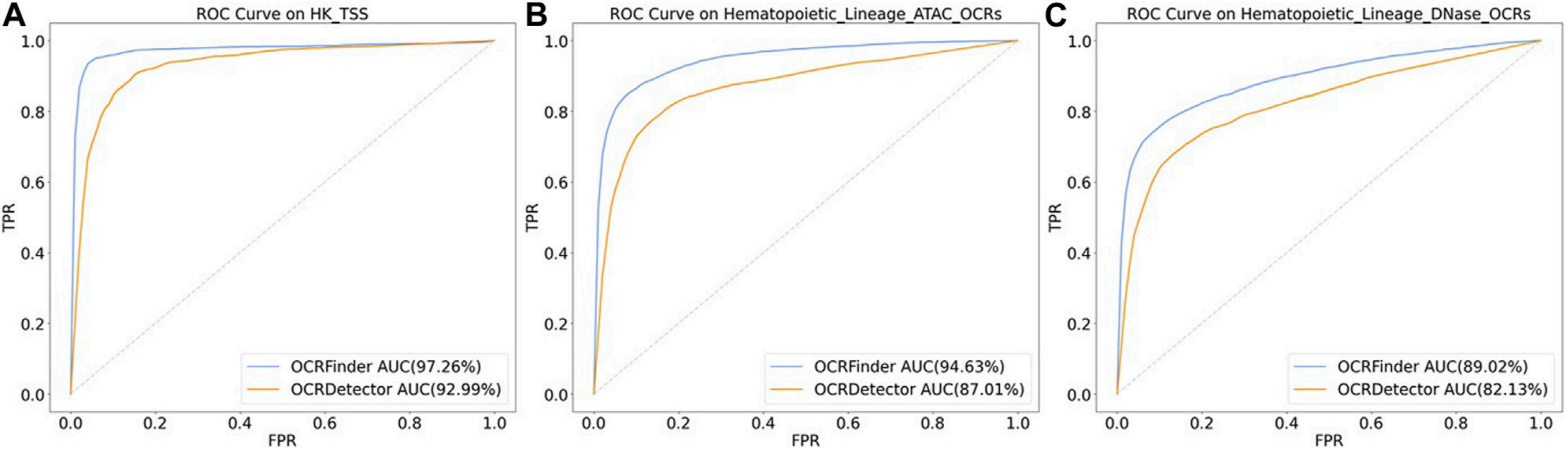
The ROC curves of OCRFinder and OCRDetector’s classifier using different test sets. **(A)** The ROC curves of OCRFinder and OCRDetector’s classifier on HK_TSS test set. **(B)** The ROC curves of OCRFinder and OCRDetector’s classifier on Hematopoietic_Lineage_ATAC_OCRs test set. **(C)** The ROC curves of OCRFinder and OCRDetector’s classifier on Hematopoietic_Lineage_DNase_OCRs test set.

**FIGURE 7:**
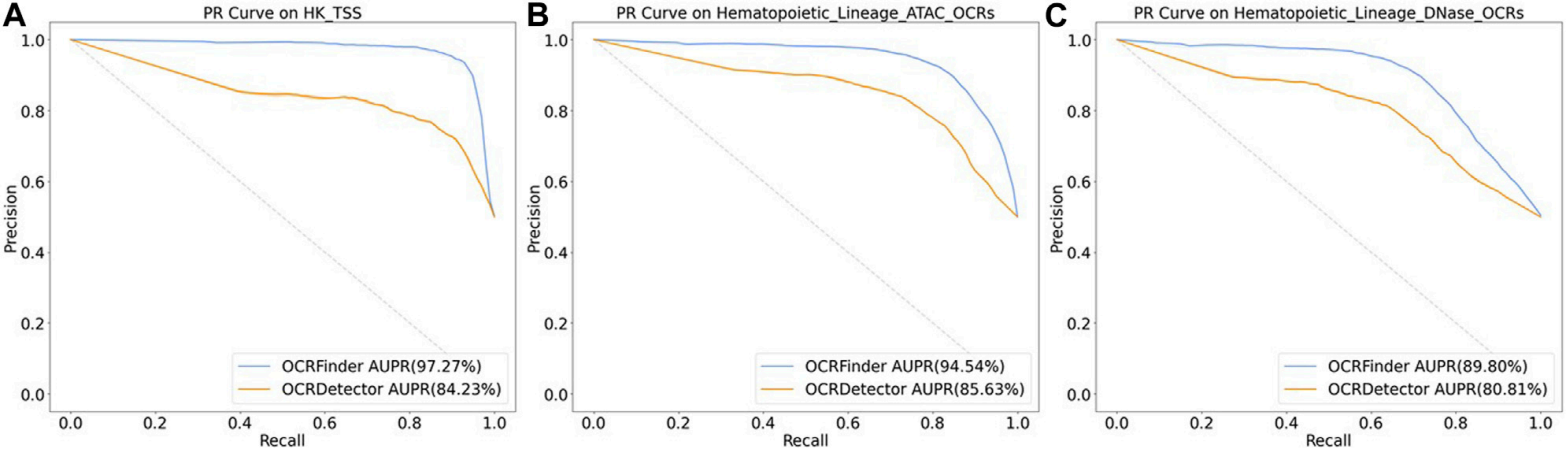
The PR curves of OCRFinder and OCRDetector’s classifier using different test sets. **(A)** The PR curves of OCRFinder and OCRDetector’s classifier on HK_TSS test set. **(B)** The PR curves of OCRFinder and OCRDetector’s classifier on Hematopoietic_Lineage_ATAC_OCRs test set. **(C)** The PR curves of OCRFinder and OCRDetector’s classifier on Hematopoietic_Lineage_DNase_OCRs test set.

We conducted comparative experiments for the classification performance test between our method and OCRDetector’s random forest classifier on AUC and AUPR metrics. Figure 6 and Figure 7 show that our method outperforms OCRDetector in both AUC and AUPR on each test set, indicating that deep learning models with noisy-label tolerance outperform traditional machine learning classifiers that rely on manually constructed features.

### 4.2 Sensitivity analysis of tissue-specific OCRs based on ATAC-seq and DNase-seq experiments

ATAC-seq and DNase-seq experiments can locate regions without nucleosomes to estimate OCRs and provide a confidence score for each OCR. We can evaluate the performance of OCRFinder by analyzing the sensitivity of OCRFinder on the OCRs obtained by the ATAC-seq or DNase-seq experiment. The sensitivity represents the overlap percentage of OCRs obtained from OCRFinder and ATAC-seq or DNase-seq experiments.

**FIGURE 8:**
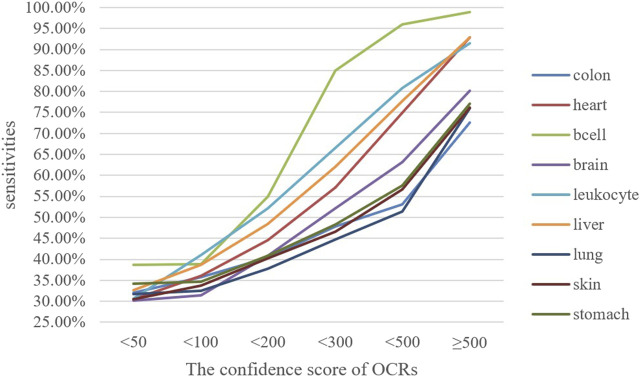
The sensitivities of OCRFinder on OCRs from ATAC-seq experiment.

Combined with the results of the ATAC-seq experiment, we selected eight tissues, including B cells, heart, brain, leukocytes, liver, lung, colon, and stomach. Among these tissues, B cells, leukocytes, and the liver are related to the hematopoietic system. They contribute most to the plasma cfDNA fragments. In contrast, the heart, brain, lung, colon, and stomach do not contribute much to cfDNA fragments. According to the confidence score provided by the ATAC-seq experiment, we divided these tissue-specific OCRs into six confidence intervals, 0–50, 50–100, 100–200, 200–300, 300–500, and above 500. Then we performed sensitivity tests on these OCRs. Figure 8 shows the sensitivity of OCRFinder at different confidence intervals, and the following conclusions can be reached:

1) With the increasing confidence score of OCRs, OCRFinder can detect more and more OCRs, and the sensitivity gradually increases from 30% to 99%. This is because OCRFinder can only detect truly opened OCRs, but the number of truly opened OCRs with low confidence levels is small. The results of OCRFinder are consistent with the results of ATAC-seq experiments.
2) OCRFinder can detect more OCRs in B cells, leukocytes, and the liver. And in these tissues, the sensitivity can vary greatly as the confidence score increases. Because these tissues contribute more to cfDNA than other tissues (Sun et al., 2019), the characteristic patterns of cfDNA fragments are greater. Therefore, OCRFinder is more sensitive to these regions.

The same conclusion can be drawn combined with the results of the DNase-seq experiment (Supplementary Figure S4):

1) The sensitivity of OCRFinder increases significantly as the confidence scores of OCRs become larger, from a minimum of 7% to an increase to a maximum of 80%.
2) At the same confidence interval, OCRFinder is more sensitive to OCRs in hematopoietic spectrum cells such as B cells, T cells, and hepatocytes.

The sensitivity results of OCRFinder in tissue-specific OCRs are highly consistent with the results of ATAC-seq and DNase-seq experiments, indicating that OCRFinder’s results are reliable.

### 4.3 Comparison experiment with other ML models

In order to verify the necessity and effectiveness of the algorithm in this paper, we also conducted comparison experiments between OCRFinder and other machine learning models. Supplementary Figure S5 and Supplementary Figure S6 show the ROC curves and PR curves of the ConvLSTM network with the OCRFinder training framework and the same network with normal training by CE loss. It can be seen that the AUC and AUPR of the model with the OCRFinder training framework are higher than the results of the normal trained model, which illustrates the effectiveness of OCRFinder training.

Table 1 shows the sensitivity results of the model with the OCRFinder training framework and the same model with normal CE-loss training. The sensitivities of deep learning models using the OCRFinder training framework in housekeeping genes and hematopoietic Lineage are higher than those of the normally trained models with CE Loss. In the non-genetic regions, models with the OCRFinder training framework have lower sensitivities. Because gene expression is not present in the non-genetic regions and OCRs are rare, the number of OCRs detected in these regions should be as low as possible. The results show that OCRFinder can have lower false positives while maintaining high sensitivity.

**TABLE 1:** The sensitivity results of different ML models.

Models	OCRs of non-genetic regions	OCRs of HK_TSS	OCRs of hematopoietic lineage by ATAC-seq	OCRs of hematopoietic lineage by DNase-seq
CNN-CE loss	7.03	93.77	79.88	69.20
CNN-OCRFinder	**6.47**	**95.12**	**81.22**	**69.81**
ConvLSTM-CE loss	12.08	92.20	79.94	68.94
ConvLSTM-OCRFinder	**7.33**	**93.71**	**80.52**	**69.28**
SVM	11.02	**94.04**	**81.76**	**70.75**
SVM-CNN-Encoder	**7.48**	93.93	80.47	69.60
RandomForest	9.77	**94.31**	**81.40**	**69.20**
RandomForest-CNN-Encoder	**6.49**	93.98	79.58	68.27
OCRFinder-base-CE loss	6.39	94.31	81.26	71.19
OCRFinder	**6.09**	**95.01**	**82.5**	**71.31**

The bold text represents the better value in each column, for each two rows.

As shown in Table 2, the AUC and AUPR of deep learning models using the OCRFinder training framework are higher than the results of the normally trained model with CE loss, indicating models with the OCRFiner training framework have better classification performance.

**TABLE 2:** The AUC and AURP results of different ML models.

Models	OCRs of HK_TSS		OCRs of hematopoietic lineage by ATAC-seq		OCRs of hematopoietic lineage by DNase-seq	
	AUC(%)	AUPR(%)	AUC(%)	AUPR(%)	AUC(%)	AUPR(%)
CNN-base with CE loss	97.09	97.53	93.56	93.74	88.09	89.27
CNN-OCRFinder	**97.43**	**97.89**	**94.15**	**94.07**	**88.59**	**89.39**
ConvLSTM-base with CE loss	94.80	91.62	90.48	88.71	84.84	83.83
ConvLSTM-OCRFinder	**97.14**	**95.27**	**92.81**	**92.42**	**87.13**	**87.59**
SVM	**96.55**	92.34	91.69	90.93	**85.58**	85.50
SVM-CNN-Encoder	96.42	**97.13**	**92.58**	**93.25**	81.11	**88.64**
RandomForest	96.27	91.80	90.82	90.67	83.70	84.89
RandomForest-CNN-Encoder	**96.99**	**96.88**	**93.04**	**93.49**	**87.27**	**89.08**
OCRFinder-base	97.02	96.88	92.72	93.47	87.37	89.31
OCRFinder	**97.26**	**97.27**	**94.63**	**94.54**	**89.02**	**89.80**

The bold text represents the better value in each column, for each two rows.

In addition, according to (Snyder et al., 2016; Sun et al., 2019; Ulz et al., 2019; Wang et al., 2021), sequencing coverage, WPS score, and the density of cfDNA heads and tails were used as four features and trained with traditional machine learning models to identify OCRs and compared with the same models using 300 features extracted by CNN. The results showed that the manually constructed features were slightly better than those extracted by CNN in sensitivity (Table 1), which may be due to the fact that CNN extracted too many features and the information was redundant. These four features are obtained through reliable experimental observation and analysis. Therefore, to further improve the classification ability of models, these four features were added together as an additional input to the network to obtain the final OCRFinder. The final OCRFinder model shows superior performance in sensitivity, AUC, and AUPR metrics (Table 1; Table 2).

To further explore the classification ability of OCRFinder, we conducted comparison experiments on the classification boundaries for OCRFinder and its base model. The base model is normally trained by CE loss and shares the same network structure as OCRFinder. The network uses the sigmoid function as the activation function in the last layer of the model, outputs a probability score of the opening level of each OCR, and ranks them from smallest to largest based on these scores. Supplementary Figure S7 shows the results of OCR detection in housekeeping genes and hematopoietic genealogies by using OCRFinder and its base model. It can be seen that OCRFinder can better suppress chromatin closed regions and improve the estimation scores of OCRs, thus suppressing false positives and improving sensitivity, implying OCRFinder can obtain sharper, clearer, and more definite classification boundaries, especially when the noise rate is greater, i.e., in the chromatin open regions of hematopoietic lineages obtained from ATAC-seq experiments.

### 4.4 Comparison experiment with other noisy-label-learning algorithms

To evaluate the noisy-label-learning ability of OCRFinder in the OCR-estimation problem, we selected several representative noisy-label-learning algorithms in the image-classification field, applied them to the OCR-estimation problem, and conducted comparative experiments with OCRFinder.

Table 3 shows the results of the comparison experiments. OCRFinder can show superior performance both in the housekeeping-gene regions and hematopoietic-genealogy regions, while other noisy-label learning algorithms have poor classification performance on OCR data, which we analyze may be due to the following reasons:

1) Co-teaching, Sel-CL, and DivideMix are sample-selection methods, but they cannot be directly used in the OCR estimation problem. Co-teaching only selects clean samples for updating models and does not use dirty samples well. For Sel-CL and DivideMix, the calculation of division thresholds does not take into account the characteristics of OCR data.
(2) ERL is a loss-sensitive method. However, its regularization method has limitations and is only suitable for more complex image datasets, and will still overfit to noisy labels in OCR data.

**TABLE 3:** The AUC and AUPR results of different noisy-label-learning algorithms.

Algorithms	OCRs of HK_TSS		OCRs of hematopoietic lineage by ATAC-seq		OCRs of hematopoietic lineage by DNase-seq	
	AUC(%)	AUPR(%)	AUC(%)	AUPR(%)	AUC(%)	AUPR(%)
Co-teaching Han et al. (2018)	96.12	93.13	90.18	88.99	81.34	85.18
ERL Liu et al. (2020)	88.77	84.02	85.41	82.88	80.49	78.34
DivideMix Li et al. (2020)	63.89	77.40	63.24	77.53	61.96	76.41
Sel-CL Li et al. (2022)	50.42	75.38	50.20	74.94	50.15	74.90
CNN-OCRFinder	**97.43**	**97.89**	**94.15**	**94.07**	**88.59**	**89.39**

The bold text represents the maximum value of each column.

### 4.5 Ablation study

We have studied the effects of removing different components to provide insights into what makes OCRFinder successful. Specifically, we tested the effects of.

• The base model with standard CE-loss training.
• The OCRFinder model using only CE loss in the pre-training stage.
• The OCRFinder model without the co-training strategy.
• The OCRFinder model without the ensemble strategy.
• The OCRFinder model without adopting the semi-supervised strategy.

The results are shown in Table 4, where we find that each component contributes to the performance of OCRFinder. The overfitting of noisy labels in the pre-training period will affect the following sample selection in the training period. Co-training of two models can avoid the overfitting caused by a single model in data cleaning and model training. Ensemble learning can strengthen the results of data cleaning. And semi-supervised learning can make full use of the entire training set.

**TABLE 4:** The AUC and AUPR results of different methods.

Methods	OCRs of HK_TSS		OCRs of hematopoietic lineage by ATAC-seq		OCRs of hematopoietic lineage by DNase-seq	
	AUC(%)	AUPR(%)	AUC(%)	AUPR(%)	AUC(%)	AUPR(%)
Base model with CE Loss	97.02	96.88	92.72	93.47	87.37	89.31
Pretraining with CE Loss	97.21	96.98	94.47	94.28	88.93	89.61
OCRFinder without co-training	97.18	96.45	94.23	94.11	88.69	89.44
OCRFinder without ensemble learning	97.25	95.85	94.22	93.84	88.65	89.15
OCRFinder without semi-supervised learning	96.86	95.20	94.15	93.70	88.57	88.92
OCRFinder	**97.26**	**97.27**	**94.63**	**94.54**	**89.02**	**89.80**

The bold text represents the maximum value of each column.

## 5 Discussion and conclusion

Feature construction is the bottleneck of OCR estimation problem, and the existing methods are based on manual construction of features, which is subjective and limited. Automatic feature extraction and classification based on neural networks will face the problem of noisy labels in OCR dataset for its dynamic change characteristics.

This paper proposes open chromatin region finder (OCRFinder), which mainly improves the model’s performance by addressing the interference of noisy labels. The key idea of OCRFinder is to integrate ensemble and semi-supervised learning into noisy label learning to obtain robustness to noisy labels, considering the characteristics of cfDNA-seq data. These improved components have proven their necessity and effectiveness in ablation experiments. Specifically, compared to OCRDetector, OCRFinder has higher sensitivity of 95.01% and 82.50% in highly expressed housekeeping genes and hematopoietic spectrum regions, while it has lower false positives of 6.08% in non-genetic regions. Compared with the classifier of OCRDetector, OCRFinder was able to obtain higher AUC and AUPR results on the ROC curve and PR curve. Moreover, the sensitivity estimation in tissue-specific regions conforms to the results of OCR confidence obtained by ATAC-seq and DNase-seq experiments. Besides, the comparative experiments with other noisy label learning algorithms illustrate that noisy label learning in the field of machine learning cannot be directly applied to the chromatin open region estimation problem, and the proposed OCRFinder is necessary. The idea of OCRFinder can also be applied to the study of solving label-unknown or label-ambiguous problems in other bioinformatics fields, providing a promising idea for solving costly annotation problems for biomedical samples.

Different cancers have different nucleosome arrangement patterns, cancer-related OCRs has different patterns in different cancers (Sun et al., 2019). The current OCRFinder is a binary classification model for discriminating the presence of OCR. In the future, we need to improve the OCRFinder into a regression model for estimating the degree of OCR openness to provide help and support for cancer analysis.

## Data Availability

The original contributions presented in the study are included in the article/Supplementary Material, further inquiries can be directed to the corresponding author.

## References

[B1] Arazo E. Ortego D. Albert P. O’Connor N. McGuinness K. “Unsupervised label noise modeling and loss correction,” in Proceedings of the International conference on machine learning, May 2019 (PMLR), 312–321.

[B2] Buenrostro J. D. Wu B. Chang H. Y. Greenleaf W. J. (2015). ATAC‐seq: A method for assaying chromatin accessibility genome‐wide. Curr. Protoc. Mol. Biol. 109 (1), 21. 10.1002/0471142727.mb2129s109 PMC4374986 25559105

[B3] Campbell J. C. Hindle A. Stroulia E. (2015). “Latent dirichlet allocation: Extracting topics from software engineering data,” in The art and science of analyzing software data (USA: Morgan Kaufmann), 139–159.

[B4] Chen P. Liao B. B. Chen G. Zhang S. “Understanding and utilizing deep neural networks trained with noisy labels,” in Proceedings of the International Conference on Machine Learning, May 2019 (PMLR), 1062–1070.

[B5] Corces M. R. Granja J. M. Shams S. Louie B. H. Seoane J. A. Zhou W. (2018). The chromatin accessibility landscape of primary human cancers. Science 362 (6413), eaav1898. 10.1126/science.aav1898 30361341 PMC6408149

[B6] Flavahan W. A. Gaskell E. Bernstein B. E. (2017). Epigenetic plasticity and the hallmarks of cancer. Science 357 (6348), eaal2380. 10.1126/science.aal2380 28729483 PMC5940341

[B7] Han B. Yao Q. Yu X. Niu G. Xu M. Hu W. (2018). Circular RNA and its mechanisms in disease: From the bench to the clinic. Adv. neural Inf. Process. Syst. 187, 31–44. 10.1016/j.pharmthera.2018.01.010 29406246

[B8] Han J. Luo P. Wang X. “Deep self-learning from noisy labels,” in Proceedings of the IEEE/CVF international conference on computer vision, 2019, 5138–5147.

[B9] Huang J. Qu L. Jia R. Zhao B. “O2u-net: A simple noisy label detection approach for deep neural networks,” in Proceedings of the IEEE/CVF international conference on computer vision, Seoul, Korea (South), November 2019, 3326–3334.

[B10] Jiang L. Zhou Z. Leung T. Li L. J. Fei-Fei L. “Mentornet: Learning data-driven curriculum for very deep neural networks on corrupted labels,” in Proceedings of the International conference on machine learning, July 2018 (PMLR), 2304–2313.

[B11] Jiang P. Sun K. Tong Y. K. Cheng S. H. Cheng T. H. Heung M. M. (2018). Preferred end coordinates and somatic variants as signatures of circulating tumor DNA associated with hepatocellular carcinoma. Proc. Natl. Acad. Sci. 115 (46), E10925–E10933. 10.1073/pnas.1814616115 30373822 PMC6243268

[B12] Klemm S. L. Shipony Z. Greenleaf W. J. (2019). Chromatin accessibility and the regulatory epigenome. Nat. Rev. Genet. 20 (4), 207–220. 10.1038/s41576-018-0089-8 30675018

[B13] Li J. Socher R. Hoi S. C. (2020). Dividemix: Learning with noisy labels as semi-supervised learning. *arXiv preprint arXiv:2002.07394* .

[B14] Li S. Xia X. Ge S. Liu T. “Selective-supervised contrastive learning with noisy labels,” in Proceedings of the IEEE/CVF Conference on Computer Vision and Pattern Recognition, March 2022, 316–325.

[B15] Liu S. Niles-Weed J. Razavian N. Fernandez-Granda C. (2020). Early-learning regularization prevents memorization of noisy labels. Adv. neural Inf. Process. Syst. 33, 20331–20342.

[B16] Lorzadeh A. Bilenky M. Hammond C. Knapp D. J. Li L. Miller P. H. (2016). Nucleosome density ChIP-Seq identifies distinct chromatin modification signatures associated with MNase accessibility. Cell Rep. 17 (8), 2112–2124. 10.1016/j.celrep.2016.10.055 27851972

[B17] Ma X. Huang H. Wang Y. Romano S. Erfani S. Bailey J. “Normalized loss functions for deep learning with noisy labels,” in Proceedings of the International conference on machine learning, November 2020 (PMLR), 6543–6553.

[B18] Mieczkowski J. Cook A. Bowman S. K. Mueller B. Alver B. H. Kundu S. (2016). MNase titration reveals differences between nucleosome occupancy and chromatin accessibility. Nat. Commun. 7 (1), 11485. 10.1038/ncomms11485 27151365 PMC4859066

[B19] Minnoye L. Marinov G. K. Krausgruber T. Pan L. Marand A. P. Secchia S. (2021). Chromatin accessibility profiling methods. Nat. Rev. Methods Prim. 1 (1), 10. 10.1038/s43586-020-00008-9 PMC10895463 38410680

[B20] Natarajan A. Yardımcı G. G. Sheffield N. C. Crawford G. E. Ohler U. (2012). Predicting cell-type–specific gene expression from regions of open chromatin. Genome Res. 22 (9), 1711–1722. 10.1101/gr.135129.111 22955983 PMC3431488

[B21] Northcutt C. Jiang L. Chuang I. (2021). Confident learning: Estimating uncertainty in dataset labels. J. Artif. Intell. Res. 70, 1373–1411. 10.1613/jair.1.12125

[B22] Quang D. Xie X. (2016). DanQ: A hybrid convolutional and recurrent deep neural network for quantifying the function of DNA sequences. Nucleic acids Res. 44 (11), e107. 10.1093/nar/gkw226 27084946 PMC4914104

[B23] Reed S. Lee H. Anguelov D. Szegedy C. Erhan D. Rabinovich A. (2014). Training deep neural networks on noisy labels with bootstrapping. arXiv preprint arXiv:1412.6596.

[B24] Ren M. Zeng W. Yang B. Urtasun R. “Learning to reweight examples for robust deep learning,” in Proceedings of the International conference on machine learning, July 2018 (PMLR), 4334–4343.

[B25] Shen Y. Sanghavi S. “Learning with bad training data via iterative trimmed loss minimization,” in Proceedings of the International Conference on Machine Learning, May 2019 (PMLR), 5739–5748.

[B26] Shin H. M. Kim G. Kim S. Sim J. H. Choi J. Kim M. (2021). Chromatin accessibility of circulating CD8+ T cells predicts treatment response to PD-1 blockade in patients with gastric cancer. Nat. Commun. 12 (1), 975. 10.1038/s41467-021-21299-w 33579944 PMC7881150

[B27] Snyder M. W. Kircher M. Hill A. J. Daza R. M. Shendure J. (2016). Cell-free DNA comprises an *in vivo* nucleosome footprint that informs its tissues-of-origin. Cell 164 (1-2), 57–68. 10.1016/j.cell.2015.11.050 26771485 PMC4715266

[B28] Song H. Kim M. Park D. Lee J. G. (2019). How does early stopping help generalization against label noise? arXiv preprint arXiv:1911.08059.

[B29] Sun K. Jiang P. Cheng S. H. Cheng T. H. Wong J. Wong V. W. (2019). Orientation-aware plasma cell-free DNA fragmentation analysis in open chromatin regions informs tissue of origin. Genome Res. 29 (3), 418–427. 10.1101/gr.242719.118 30808726 PMC6396422

[B30] Toneva M. Sordoni A. Combes R. T. D. Trischler A. Bengio Y. Gordon G. J. (2018). An empirical study of example forgetting during deep neural network learning. *arXiv preprint arXiv:1812.05159* .

[B31] Ulz P. Perakis S. Zhou Q. Moser T. Belic J. Lazzeri I. (2019). Inference of transcription factor binding from cell-free DNA enables tumor subtype prediction and early detection. Nat. Commun. 10 (1), 4666. 10.1038/s41467-019-12714-4 31604930 PMC6789008

[B32] Ulz P. Thallinger G. G. Auer M. Graf R. Kashofer K. Jahn S. W. (2016). Inferring expressed genes by whole-genome sequencing of plasma DNA. Nat. Genet. 48 (10), 1273–1278. 10.1038/ng.3648 27571261

[B33] Wang J. Chen L. Zhang X. Tong Y. Zheng T. (2021). OCRDetector: Accurately detecting open chromatin regions via plasma cell-free DNA sequencing data. Int. J. Mol. Sci. 22 (11), 5802. 10.3390/ijms22115802 34071577 PMC8198695

[B34] Wang Y. Lai X. Wang J. Xu Y. Zhang X. Zhu X. (2022). A joint model considering measurement errors for optimally identifying tumor mutation burden threshold. Front. Genet. 13, 915839. 10.3389/fgene.2022.915839 35991549 PMC9386083

[B36] Wang Y. Wang J. Fang W. Xiao X. Wang Q. Zhao J. (2023). TMBserval: a statistical explainable learning model reveals weighted tumor mutation burden better categorizing therapeutic benefits. Front. Immunol. 14, 1151755. 10.3389/fimmu.2023.1151755 37234148 PMC10208409

[B35] Wang Z. Tu K. Xia L. Luo K. Luo W. Tang J. (2019). The open chromatin landscape of non-small cell lung carcinoma. Cancer Res. 79 (19), 4840–4854. 10.1158/0008-5472.CAN-18-3663 31209061

