# Supplementary material for: OCRFinder: a noise-tolerance machine learning method for accurately estimating open chromatin regions
Source: Front Genet. 2023 Jun 1;14:1184744. doi: 10.3389/fgene.2023.1184744 (PMC10267440; doi:10.3389/fgene.2023.1184744)
Supplement: Supplementary file 1 [file Image1.pdf]

Jiayi Ren[1,2], Yuqian Liu[1,2,†], Xiaoyan Zhu[1,2], Xuwen Wang[1,2], Yuxin Liu[1,2], Wenqing Hu[1,2], Xuanping Zhang[1,2], Jiayin Wang[1,2,*]

[1]School of Computer Science and Technology, Xi'an Jiaotong University, Xi'an 710049, China

[2]Shaanxi Engineering Research Center of Medical and Health Big Data, Xi'an Jiaotong University, Xi'an 710049, China

[†]These authors have contributed equally to this work and share the first authorship

*** Correspondence:**

Jiayin Wang, wangjiayin@mail.xjtu.edu.cn

Supplementary Material

OCRFinder: A Noise-tolerance Machine Learning Method for Accurately Estimating Open Chromatin Regions

1 Supplementary Figures

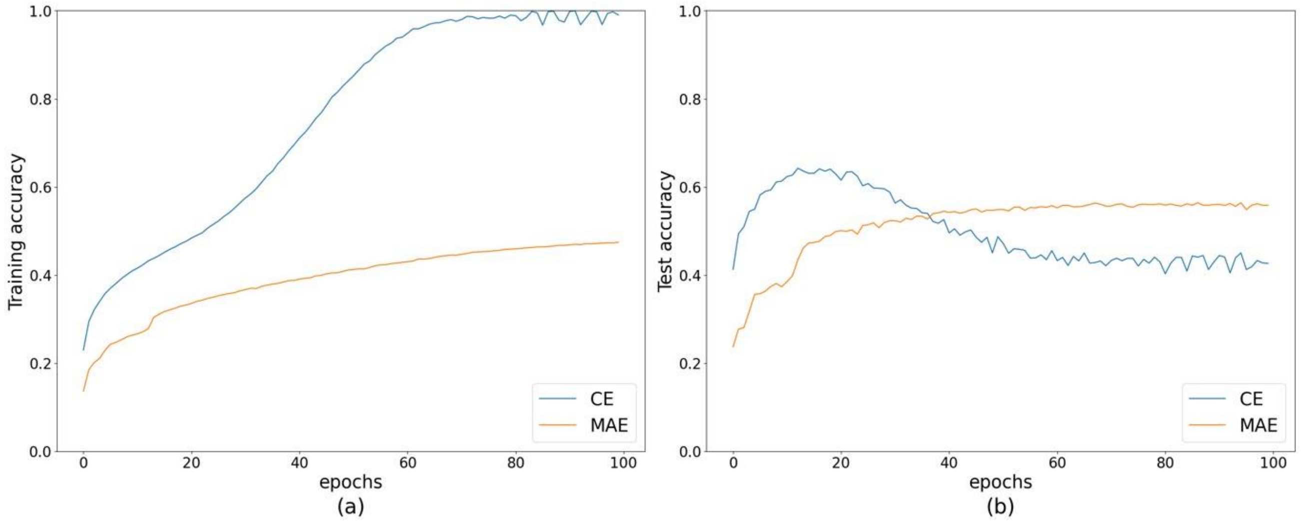

Supplementary Figure 1. The accuracy under CE loss and MAE loss. (a) Accuracy on the training set. (b) Accuracy on the test set.

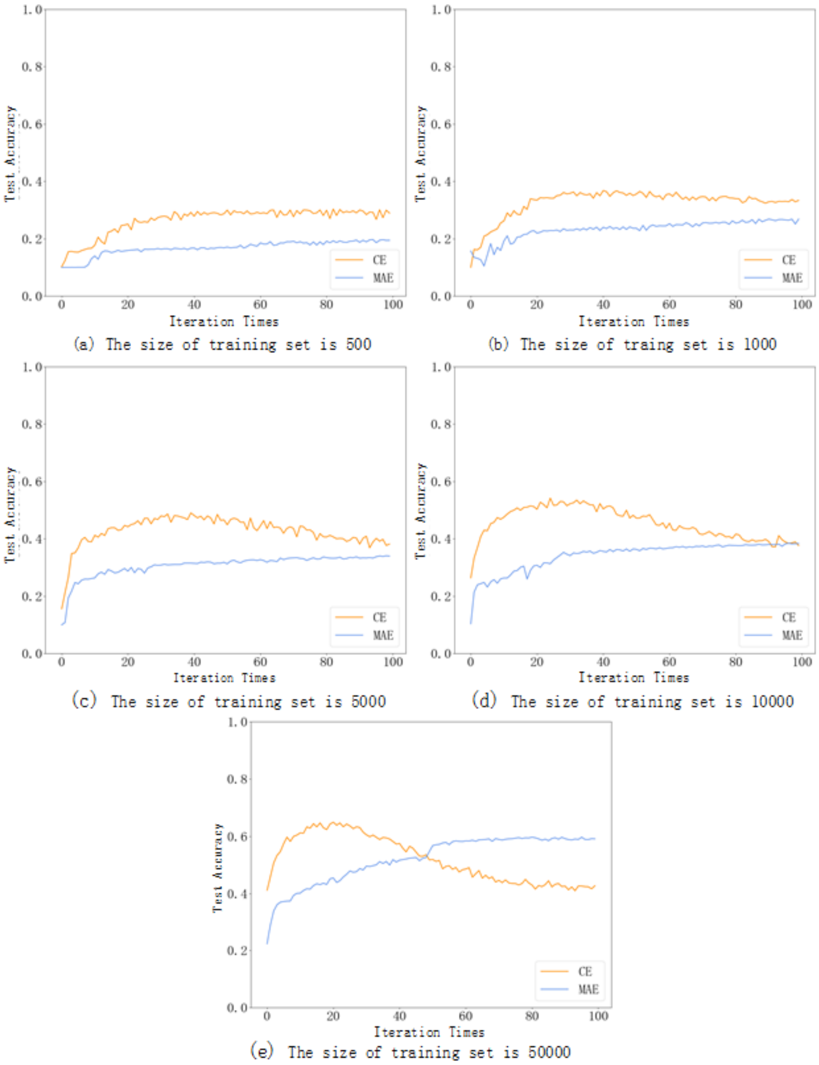

Supplementary Figure 2. Test accuracy of models with different dataset sizes.

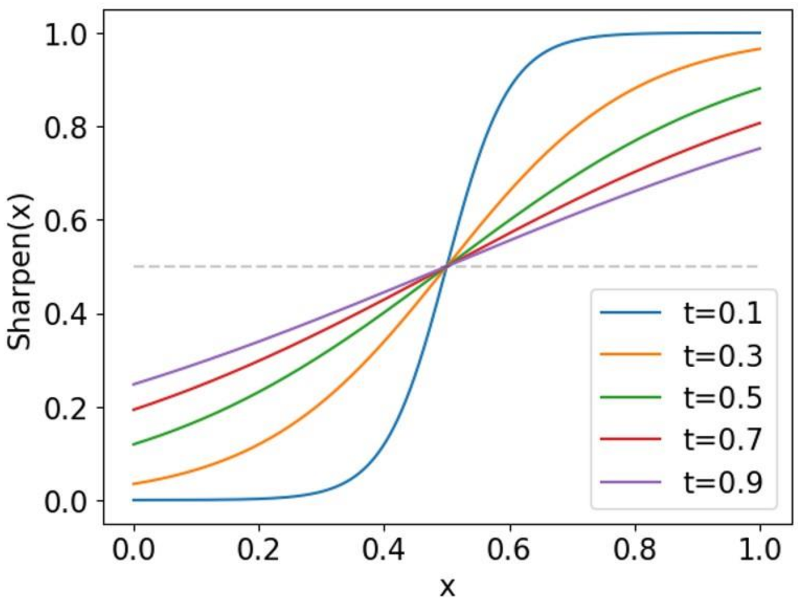

Supplementary Figure 3. The effect of Sharpen function.

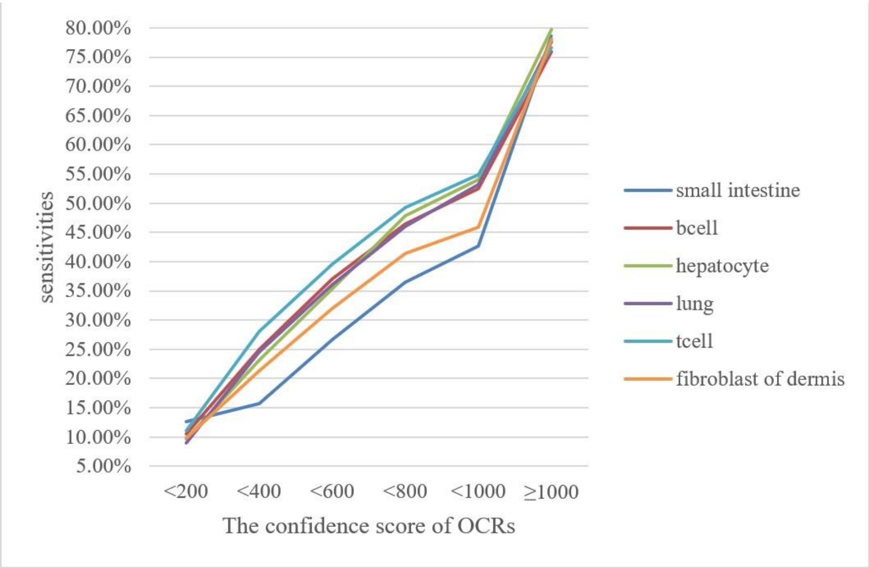

Supplementary Figure 4. The sensitivities of OCRFinder on OCRs from DNase-seq experiment.

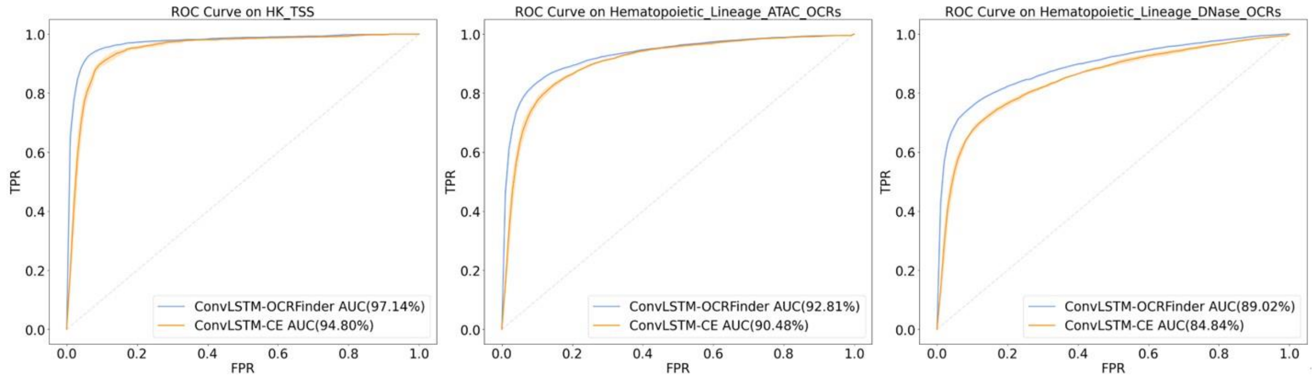

Supplementary Figure 5. The ROC curves of ConvLSTM models with OCRFinder training framework and models trained with CE loss using different test sets. (a) The ROC curves on HK_TSS test set. (b) The ROC curves on Hematopoietic_Lineage_ATAC_OCRs test set. (c) The ROC curves on Hematopoietic_Lineage_DNase_OCRs test set.

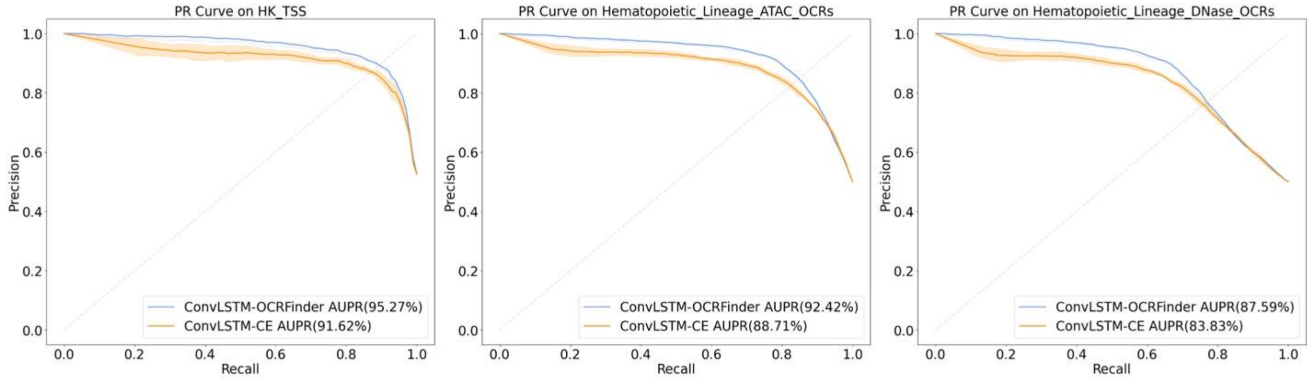

Supplementary Figure 6. The PR curves of ConvLSTM models with OCRFinder training framework and models trained with CE loss using different test sets. (a) The PR curves on HK_TSS test set. (b) The PR curves on Hematopoietic_Lineage_ATAC_OCRs test set. (c) The PR curves on Hematopoietic_Lineage_DNase_OCRs test set.

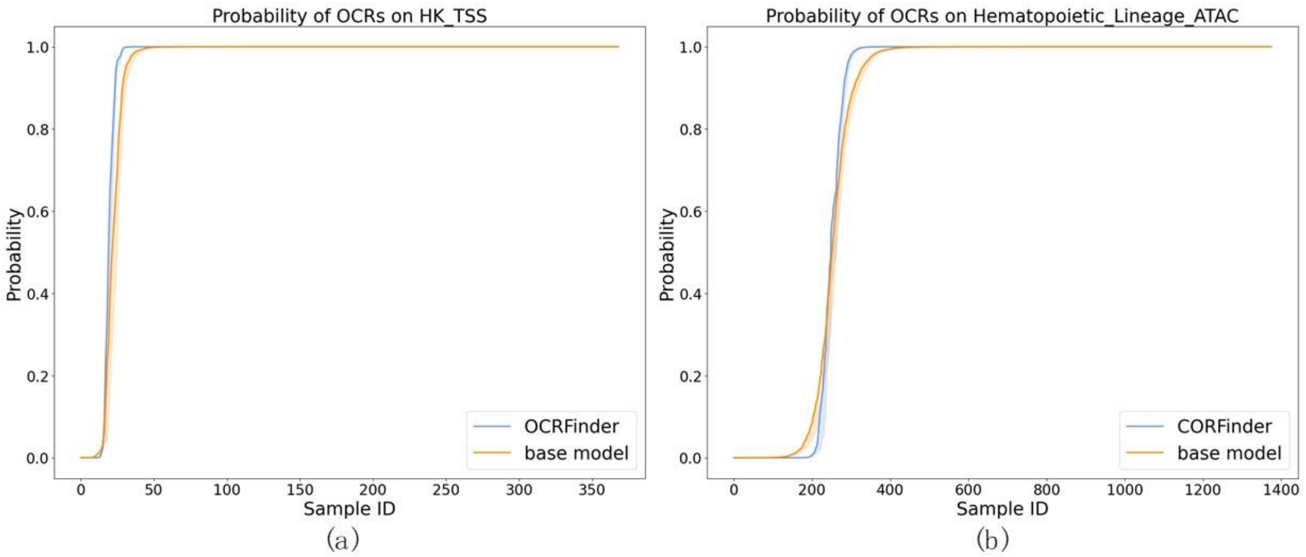

Supplementary Figure 7. The comparison with base model.（a）Comparison within the OCR of housekeeping genes. (b) Comparison within the OCR of ATAC-seq experiment.
